# Supplementary material for: Tough double-bouligand architected concrete enabled by robotic additive manufacturing
Source: Nat Commun. 2024 Aug 29;15:7498. doi: 10.1038/s41467-024-51640-y (PMC11362293; doi:10.1038/s41467-024-51640-y)
Supplement: Supplementary file 1 — Supplementary Information [file 41467_2024_51640_MOESM1_ESM.pdf]

Supplementary Information

Tough Double-Bouligand Architected Concrete Enabled by Robotic Additive Manufacturing

Arjun Prihar[1], Shashank Gupta[1], Hadi S. Esmaeeli[1], Reza Moini[1*]
[1] Department of Civil and Environmental Engineering, Princeton University, Princeton, NJ, USA

[*] Corresponding Author Email Address: Reza.moini@princeton.edu

Supplementary Note 1: One-Component and Two-Component Additive Manufacturing Process

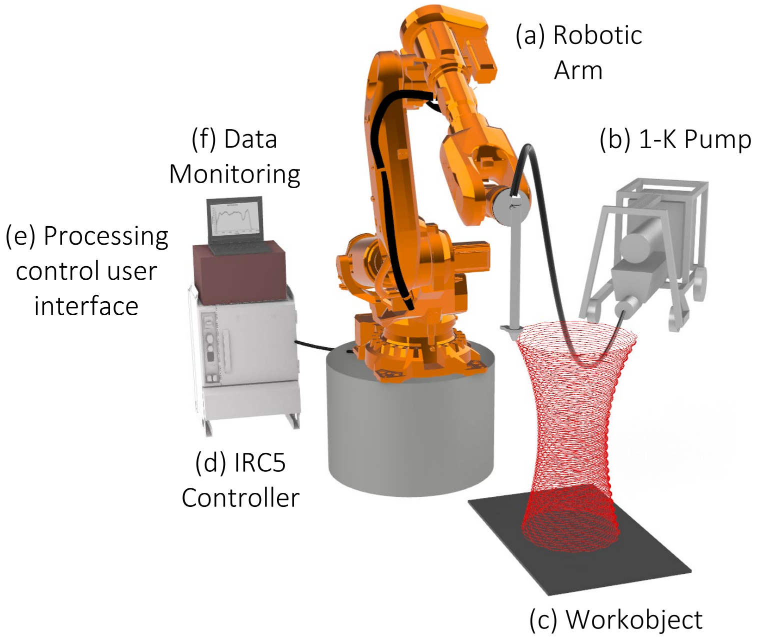

Supplementary Fig. 1. Schematic of one-component (1-K) process: (a) ABB IRB 4600 robot arm (1.65 m reach, 60 kg payload), (b) Concrete one-pot feedstock, (c) Workobject with visualized toolpath, (d) ABB IRC5 controller, (e) Processing control user interface, (f) Monitoring real-time temperature and pressure sensor read-outs.

One-Component Additive Manufacturing Process. A basic concrete additive manufacturing process requires a feedstock to supply concrete through a nozzle end-effector guided by a robot arm along a digitally predefined path. A schematic depiction of a 1-K process is shown in Supplementary Fig. 1 with an ABB IRB 4600 robot arm (1.65 m reach, 60 kg payload) (Supplementary Fig. 1a). The concrete is continuously mixed, and continuously fed into a cavity pump (Supplementary Fig. 1b) that conveys material to a nozzle end-effector via a flexible hose of 25 mm internal diameter. A workpiece (Supplementary Fig. 1c) is positioned in front of the ABB IRB 4600 robot arm with a predefined toolpath comprised of discrete, sequential points. The robot arm and the end-effector position are defined relative to a global reference frame, termed as the Workobject. The Workobject can be defined in the programming language such as RAPID or KRL used to control ABB or KUKA industrial robots, respectively. The RAPID code for any given toolpath is executable by ABB's Industrial Robot Controller (IRC5) (Supplementary Fig. 1d).

The pressure and temperature sensors were outfitted throughout the 1-K platform. Pressure sensors have a range of 0 to 40 bar and temperature sensors had a range of -40°C to 150°C. Temperature sensors are strategically positioned at the pump and nozzle outlets to allow for the monitoring of the material consistency and the operational aspects of the extrusion process. Pressure sensors are positioned at the pump outlet and the nozzle inlet to evaluate changes in baseline extrusion pressure. The presence of entrapped air at the mixing and batching stage can result in temporarily lower pressures owing to the air's compressibility. Slight variations in the material composition may result in pressure inconsistencies. The

thixotropic structural build-up from the evolution of the material as a function of residence time in the 1-K pump hopper and hose can result in locally or globally higher pressures.

The temperature sensor fitted at the end of the nozzle tip allows for both an instantaneous indication of the effect of material mixing and extrusion among batches and an overall indication of change in temperature due to the thixotropic structural build-up over time. The continuous monitoring and recording of the material temperature data also acts as an indirect analysis for consistency and operational issues such as potential material clogging or pumping failure due to overheating, thus providing a path for in-line troubleshooting. The digital measurements from the pressure and temperature sensors are sent to a processing control user interface (Supplementary Fig. 1e) and displayed in real time on a monitor (Supplementary Fig. 1f).

Buckling failure has been reported to be controlled by a cementitious material's stiffness (shear storage modulus, G') in 1-K extrusion processes, while yielding failure in the lower layers has been demonstrated to be correlated with a material's yield stress [1,2]. The drawback of the 1-K process is the limited ability to directly control the material composition, rheological properties, and rate of hydration at the nozzle, thus imposing constrains with respect to geometrical fidelity and building rates [1,3,4]. On the other hand, the 1-K process provide a facile approach for one-pot extrusion of a varieties of cement-based and alternative binders [5–8].

Two-Component Additive Manufacturing Process. The two-component additive process is differentiated from the one-component process as it allows for near real-time tuning of the material's rheological properties through the acceleration or deceleration of hydration at the nozzle [9,10]. Unlike a 1-K process that relies on the initial, continuous mixing and pumping of the material in a single stage [1,11], the 2-K process includes a continuous dry powder feedstock followed by mixing and two-stage pumping, and a secondary agitation chamber within the nozzle end-effector featuring an additional inlet for a liquid-phase solution such as a chemical admixture [19,26,64,66]. Prior to the extrusion, a set-retarder may be mixed into the concrete to either increase the open time of the material or alleviate the required pumping pressure [10]. A set-accelerant intermixed immediately prior to extrusion within the nozzle end-effector works to reverse the effect of any upstream retarders from the batch mixing stage as well as rapidly increase the rate of calcium-silicate-hydrate (C-S-H) formation in the concrete [10]. The C-S-H nucleation and bridging between cement particles is the primary mechanism for increasing the thixotropy of the material upon addition of the set-accelerant [10,12,13].

The 2-K additive manufacturing process established in this work is depicted in Fig. 2 features an ABB IRB 6700 robot arm (2.85 m reach, 150 kg payload) (Fig. 2a) situated atop an ABB IRBT 6004 track (8.5 m, with 5.7 m linear travel distance) to allow for fabrication of multiple, larger workpieces. The 2-K process leverages a two-stage pump featuring in-situ charging of dry feed and an automated supply of water to continuously mix and convey concrete to the nozzle agitation chamber (Fig. 2b). Below the mixing chamber is located an auger which pumps concrete into a progressive cavity pump that is designed for consistent extrusion of material through the hose. The liquid solution drawn from an accelerant reservoir (Fig. 2c) through a smaller progressive cavity pump feeds into to the 2-K nozzle, injecting set-accelerant into the concrete (Fig. 2d). The concrete and the injected liquid phase entered an agitation chamber within the 2-K nozzle end-effector and intermixed prior to extrusion (Fig. 2e). The agitation chamber and nozzle are mounted on the 6th axis of the robot arm (using an L-shape coupler). The agitation is achieved by a servo motor rotating a series of interlaced rods at 450 revolutions per minute to ensure homogenization of the concrete and intermixing of the liquid accelerant prior to deposition.

One or more workpieces (Fig. 2f) are positioned in front of the robot track and robot arm with the predefined toolpath for multiple objects. The end-effector is defined relative to local reference frames, termed as the Workobject in RAPID code. Workobjects are commonly defined separately for each executable toolpath. RAPID is the programming language used to control ABB industrial robots. As in the 1-K process, the RAPID code for a toolpath is executable by ABB's Industrial Robot Controller (IRC5) (Fig. 2g). The IRC5 controls all of the dry parameters, such as the end-effector geometry and Workobject local coordinate definitions, and robot movement and speed. An InterProcess Communication (IPC) (Fig. 2h) was used to control all wet parameters related to pumping pressures and flow rates in-situ.

Wet parameters were measured at various locations by sensors and displayed in real-time on a monitor (Fig. 2i) to evaluate the operational performance and material quality. Pressure sensors have a range of 0 to 40 bar, and temperature sensors have a range of -40°C to 150°C. The concrete pump outlet is fitted with a temperature and pressure sensor to observe the material as it is fed into a hose of 25 mm internal diameter. During operation, the pressure and temperature at the concrete pump ranged from 14 to 16 bar and 29°C to 32°C, respectively. Pressure sensors are located at the concrete and accelerant inlets of the agitation chamber to measure the consistent flow prior to intermixing. Lastly, the temperature is measured in the agitation chamber as well as at the nozzle tip immediately prior to extrusion.

During operation, the pressure and temperature of the agitation chamber ranged from 0.4 to 0.7 bar and 32°C to 38°C, respectively. The pressure sensors are particularly critical to providing an instantaneous indication of clogging and overall consistency of the extrusion process. The temperature within the agitation chamber is particularly important for monitoring potential issues such as overheating of material in the agitation chamber due to relatively high energy mixing and addition of set-accelerant. Spikes in either the temperature or pressure in the agitation chamber would be indicative of an operational or processing issues, and can be immediately addressed once observed by the real-time monitoring. These highly instrumented 2-K platform enable informed decision making and can be further enhanced by automating the necessary adjustments in the dry or wet processing parameters based on sensor information [26]. Communication between the IRC5 and the IPC was established using an ethernet cable to allow for the concrete and accelerant flow rates to be controlled manually through a human-machine interface (HMI) or digitally through the execution of RAPID code specifying input values for flow rates. Consequently, all fabrication parameters, from concrete and accelerant flow rates to robot path, position, and speed, can be digitally controlled for the fabrication of highly complex, architected materials. Developing communication between often segmented equipment used in additive manufacturing of concrete is necessary in order to improve upon the existing open-loop manufacturing operations that typically lead to trial and error and therefore waste of materials and resources.

Supplementary Note 2: Comparison of One-component (1-K) and Two-Component (2-K) Processes

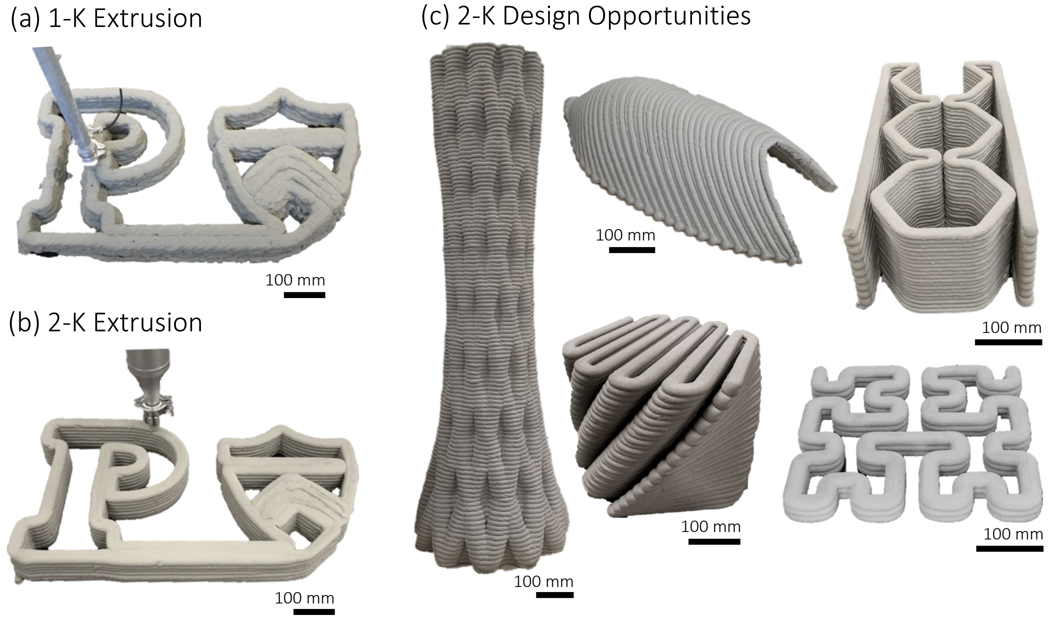

Supplementary Fig. 2. Additively manufactured structures fabricated using 1-K and 2-K robotic processes, (a) A logo additively manufactured using a 1-K extrusion process (using an IRB 4600) compared to (b) The same toolpath additively manufactured using a 2-K extrusion process (using an IRB 6700), and (c) Several design opportunities including a complex column, non-planar shell, and hexagonal, helicoidal, and Hilbert compliant geometries using the 2-K process.

The 1-K robotic additive manufacturing process is the most prevalent fabrication pathway to date and has been adopted by numerous researchers [14–16] and multiple industrial concrete additive manufacturing companies [17]. Though widely used, the 1-K process has a limited build-up rate in which the maximum height of a concrete element is constrained by the shear yield strength and stiffness of the extruded material depending on whether the yielding or buckling is the dominate mode of fresh failure [1,18–20]. Both the shear yield strength and shear stiffness gains are governed by the increase in thixotropy, which is directly proportional to the rate of formation of hydration products, specifically the precipitation of C-S-H [10]. The 1-K process, depending on the scale, requires slower printing during the manufacturing of the elements to prevent collapse and allow for the build-up to occur or intermittent pauses between fabrication of multiple elements [21], else can become tedious to be utilized as a continuous batching and feeding process. In addition, the intermittent or slower nature of 1-K process in the deposition between layers belabours the fabrication process, thereby potentially exacerbating the effect of cold joints or weak interface between the layers, respectively [21–24], or introducing additional heterogeneities among the interfaces and filaments.

The 2-K process, on the other hand, aptly addresses these issues by allowing for greater control over the material's rheological properties to increase the shear yield strength and shear stiffness of the material that is necessary for structures more than 0.5 – 1 meter tall [19]. Moreover, the ability to dose the accelerant greatly improves the geometric quality of the final objects and enables the fabrication of more

complex designs (Supplementary Fig. 2b), as compared to the 1-K process (Supplementary Fig. 2a). This, in turn, allows for the execution of intricate material architectures and ambitious purposeful designs (Supplementary Fig. 2c). In addition, the use of a liquid phase solution at the nozzle enabled in the 2-K process can allow for the extra degree of freedom in tuning the material's interfacial and filament chemistry, thus allowing the tuning and functionally grading the mechanical responses. In contrast to the 1-K process, the 2-K process allows for precise and simultaneous control of wet and dry processing parameters.

In both processes, the real-time monitoring and instantaneous communication between the various robotic and pumping equipment can help transition away from open-loop manufacturing operations that often rely on trial and error or one-off procedures.

There are additional challenges in the extrusion-based additive manufacturing of hydraulic suspensions (e.g., cementitious materials), as compared to the more commonplace additive manufacturing of thermoplastics, carbon-fiber reinforced polymers, and other ceramics These may include significant early-age deformation, partly due to the large size of the printed filaments[25], and reliance on early-stage hydration for hardening [26]. For instance, numerous studies have focused on engineering the rheological and hydration properties of additively manufactured cementitious materials to address these challenges [27–30].

Moreover, one of the major challenges in scaling up additive manufacturing technology is balancing the often large size of the structures with the fabrication rate. Large nozzle sizes have been widely developed to address this issue [31]. Previous works have discussed the effect of structure size on early-age deformation and the yield stresses required for a given production rate [32].

Two-component extrusion systems are defined by the ability to accelerate the hydration of cementitious materials at the nozzle tip. This can increase the production rate in the vertical direction in order to achieve large-scale printing [31]. In short, for effective scaling up, it is beneficial to use larger filament sizes and a 2K system. This approach not only enhances production rates but also allows for better control over feature sizes (solids or pores) within a reasonable fabrication time. This is particularly advantageous for the mechanical properties and design of mesoscale architectures in civil infrastructure components.

<u>Supplementary Note 3:</u> LEFM Analysis of Crack Twisting in Bouliganda Architecture

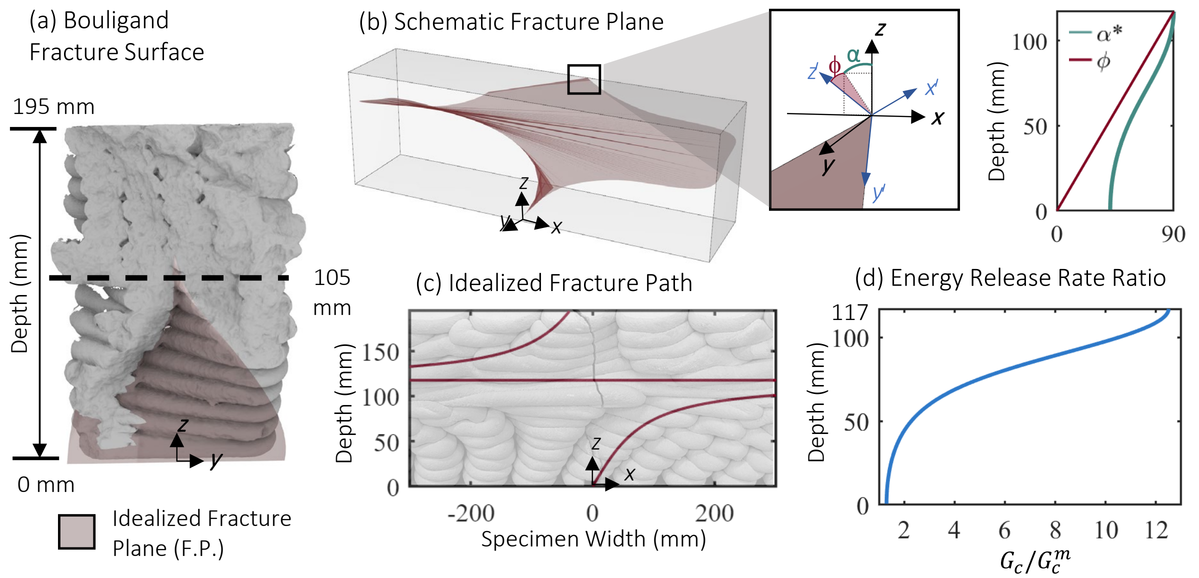

Supplementary Fig. 3. (a) Cross-sectional view of bouligand fractured plane highlighting the idealized fractured plane, (b) Schematic representation of idealized fractured plane and mathematical definition of rotated cartesian axes x', y', z', twist angle, f, kink angle, α, (c) Idealized fractured path projected on Z-X surface, and (d) Ratio between energy release rate of propagating crack, G_c, and the energy release rate of a linear elastic material under Mode-I fracture, G_c^m [33].

Supplementary Note 4: Micro-Computed Tomography of Additively Manufactured Concrete

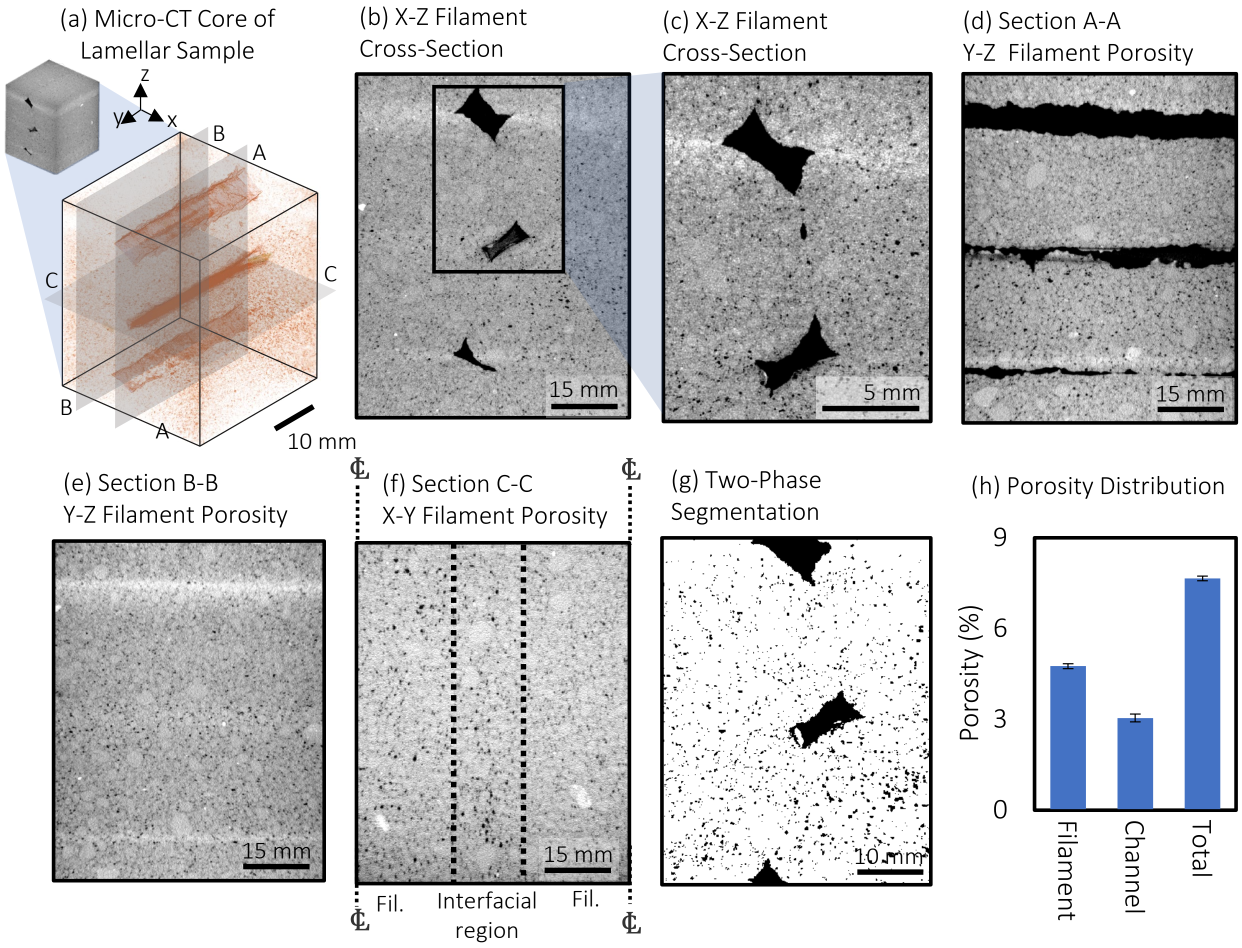

Supplementary Fig. 4. (a) Representative lamellar architecture and two dimensional cross-sectional views of the layered additively manufactured filament obtained from micro-CT, (b,c) the X-Z plane highlighting the diamond-shaped macroscopic pores and interfacial regions of locally higher anhydrous cement, (d,e) Y-Z plane highlighting regions of macroscopic pores and regions of locally higher anhydrous cement, (f) the X-Y plane highlighting the interfacial regions of locally higher porosity between adjacent filaments in the same layer. (g) A binary segmentation of the X-Z plane of the micro-CT specimen, (h) Porosity distribution obtained from the binary segmentation differentiating the total porosity contribution from the diamond-shaped macroscopic pores from surrounding filament porosity and the total porosity.

A Zeiss X-ray microscope was used to conduct micro-computed tomography (Micro-CT) and map the morphology and distribution of pore phase. A 75 mm wide × 40 mm tall × 40 mm thick, additively manufactured specimen was cut from a lamellar specimen printed at 60 mm/s speed, representative of the printing speed used for the fabrications in this study. The specimen was examined at 0.4x magnification. The acquisition was performed with a source power of 140 kV and 10 W. A total of 1601 projections were taken, with one image per rotation angle and a total rotation of 360°. Each projection required a 10 second exposure to maintain an intensity greater than 5000 counts per exposure. The average transmittance was approximately 20% through the thickness and 13% through the width. The source had a

working distance of 110 mm and the detector had a working distance of 68 mm relative to the specimen position. The scanning parameters correspond to a resolution of 42 µm.

Binary segmentation was performed on 100 cross-sectional slices taken from the micro-CT specimen at a spacing of 0.25 mm by manual thresholding of grayscale intensities using image processing toolbox of MATLAB 2022 [34]. The tangent-slope method was used to evaluate the upper threshold intensity limit of pores, which is determined as the intersection point of the tangents at the initial region of the gray-scale histogram and the upper region of the hydrated product peak. Finally, the pixel label data was obtained from the binary segmented images using MATLAB 2022 [34] to quantify the pore and solid phases.

The interfaces in the architected specimens are predominantly characterized by two main microstructural features: (i) a relatively more porous or less hydrated interfacial region and (ii) a diamond-shaped macroscopic pores at the intersection of 3D-printed filaments leading to the formation of a macro-channel (Supplementary Fig. 4a), both of which can interact with a propagating crack in architected materials. The macroscopic channels (approximately 5 mm wide) were found as a result of the slight filament offset that arose from the alternating nozzle direction along the digitally defined extrusion path (Supplementary Fig. 4b, c) as reported at the 3D-printed cement paste-scale by the authors [35,36]. A region of lower degree of hydration (i.e., higher anhydrous cement grains) was frequently observed along the interfaces at the vicinity of the macroscopic channels, shown as brighter horizontal regions (Supplementary Fig. 4b,c,e). This region was hypothesized to be a consequence of the surface drying that occurred during the time gap between printed layers, coupled with higher drying of material at the vicinity of macroscopic channels. These findings are also surprisingly reported in 3D-printed cement paste [35].

The binary segmentation was performed on 100 X-Z cross-sections to quantify the porosity in the specimen (Supplementary Fig. 4g). The contribution to the total porosity is from the diamond-shaped macro-channel and the porosity in the surrounding filament (Supplementary Fig. 4h). The total porosity in the specimen is 7.7 % ± 0.08 %, with 3.0 % ± 0.13 % arising from the presence of the channel. Hence, the channel constitutes approximately 40% of the total porosity in the lamellar specimens. It should be noted that total porosity in lamellar architecture is within the range of 5 – 13% porosity reported in the literature for similar nozzle size [37–40]. We hypothesize that this percentage decreases for architected specimens in which the filaments overlap at offset angles, thereby reducing the uniformity of the channel and ultimately reducing the total porosity. The sharp morphology of the diamond-shaped macroscopic pores or channels as observed in Supplementary Fig. 4c,g is conducive to stress concentrations and thus crack-flaw interactions.

The macroscopic pores are hypothesized to preferentially deflect the crack path, thus driving the predominant toughening mechanism of crack twisting. Additionally, the stacked formation of voids (Supplementary Fig. 4d) and the higher degree of porosity along the vertical interface between filaments (Supplementary Fig. 4c, f) may contribute to a stabilization of the propagating crack as it twists along filament interfaces. Further investigation of the heterogeneous microstructure may grant additional evidence on the role of weak interfaces on fracture response [41].

Supplementary Note 5: Architecture-Specific Toolpath Generation Algorithms Generation for Robotic Additive Manufacturing

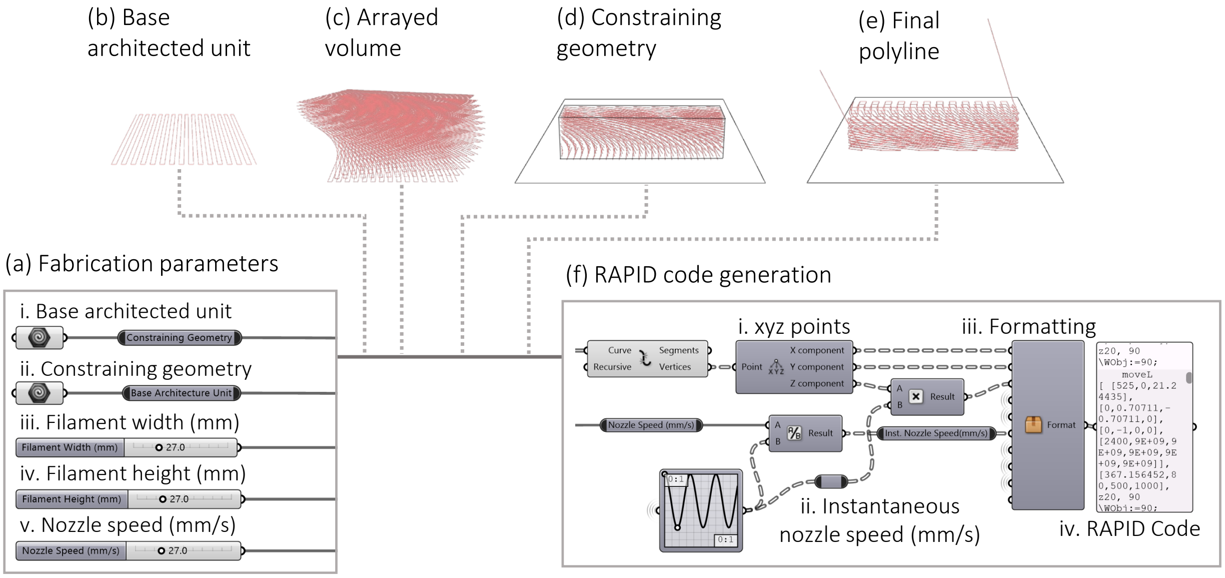

Supplementary Fig. 5. Architected toolpath generation algorithm, (a) Input parameters, including i. base architecture unit, ii. Constraining geometry, iii. filament width (mm), iv. filament height (mm), and v. nozzle speed of robot end-effector prior to adjusting for graded architectural requirements, (b) Visualization of input architecture unit, (c) Array of architecture unit to form a desired large volume, (d) Cropping of architected volume by a prescribed input geometry and positioning on Workobject, (e) Joining of discrete curves to create a continuous toolpath, (f) Generation of RAPID code, beginning with i. determining incremental x-y-z point coordinates, ii. optional computing of instantaneous speed (in this case based on a sinusoidal gradient), iii. formatting point coordinates and nozzle speed to generate executable RAPID code, and iv. final RAPID code.

Most additive manufacturing processes rely on toolpath algorithms to execute fabrication tasks. Algorithms take user-defined inputs and execute an exact series of operations to produce an output. Toolpath algorithms are used to convert a desired input geometry into a script of code that is executable by the relevant manufacturing technology, such as RAPID for ABB or KRL for KUKA. Most of the script consists of movement instructions that define target coordinates for the robot end-effector to reach before executing the next instruction. The algorithms in this study were developed in Grasshopper, a visual programming interface within Rhinoceros 3D [42].

Complex architectures, for instance functionally graded architectures, require a fabrication process that allows for the instantaneous adjustment of filament dimensions. These dimensions are most efficiently controlled by varying the nozzle speed between each Target point (Supplementary Fig. 5f-*i*). The instantaneous nozzle speed (Supplementary Fig. 5f-ii) is calculated by multiplying a user-defined mathematical function for the desired functional grading if needed (Supplementary Fig. 5f-*ii*) with the user-defined nozzle speed (Supplementary Fig. 5a-*v*) to control the total volume extruded between two Targets. The same mathematical function can also be used to scale the z-coordinate of the polyline points to meet the desired layer height. Once computed, the instantaneous speeds and cartesian coordinates of

each point can be formatted (Supplementary Fig. 5f-*iv*) as Move instructions for an entire toolpath. The robot instructions for a specific toolpath comprise a Procedure in the RAPID program, which is included within a Module along with the relevant global definitions of end-effectors and Workobjects.

The architecture-specific algorithm is agnostic to any individual robot's movement capabilities. Hence, the robot path is simulated in a virtual environment within software (RobotStudio) as a dry-run to assess for any compilation errors as well as any movement errors, such as collisions, physically unreachable points, or other singularity issues due to a current position of the joints. Individual Modules are then called in the 'main' Program file, and the entire RAPID Program inclusive of main and the respective toolpath Modules is uploaded to the IRC5 Teach Pendant. Specific toolpath Procedures can be executed individually, or as a series of consecutive prints by executing main. Once uploaded, the robotic platform is equipped to execute toolpaths to fabricate complex bio-inspired cementitious architected materials.

Supplementary Note 6: Conventional Toolpath Generation Algorithms for Robotic Additive Manufacturing

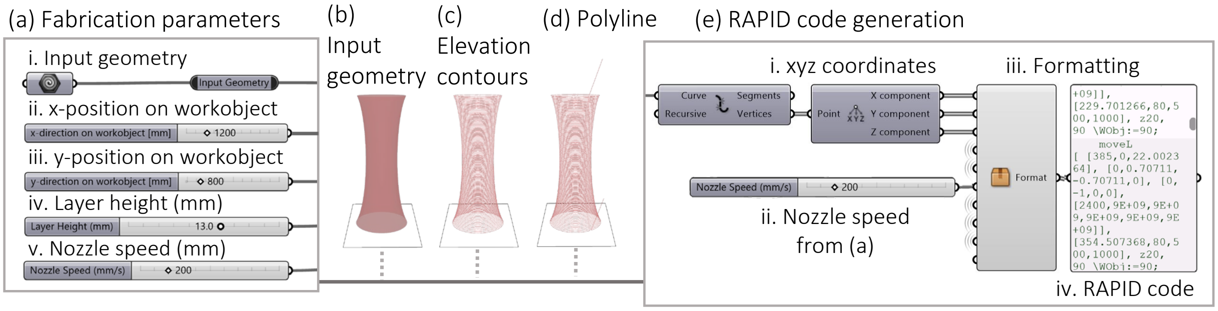

Supplementary Fig. 6. Conventional toolpath generation algorithm: (a) Input parameters, including i. input geometry, ii. x-dimension position on Workobject, iii. y-dimension position on Workobject, iv. layer height (mm), and v. speed of robot end-effector, (b) Visualization of input geometry positioned on Workobject, (c) Generation of closed-curve, elevation contours of the object, (d) Joining of discrete curves to form a continuous toolpath, (e) Generation of RAPID code, beginning with i. determining incremental x-y-z point coordinates, ii. prescribing speed, iii. formatting point coordinates and nozzle speed to generate executable RAPID code, and iv. final RAPID code.

Conventional toolpath algorithms generally require three distinct steps. Firstly, the fabrication parameters, including layer height, nozzle speed, and the object's position on the Workobject, are defined by the user (Supplementary Fig. 6a). Next, the boundary representation of a user's input geometry (Supplementary Fig. 6b) is sliced at intervals equal to the prescribed layer height, thereby creating elevation contours (Supplementary Fig. 6c). The curve is then discretized into points with associated x-y-z coordinates. The points are ordered in series, starting with the lowest contour and moving upwards. This series is used to represent the final geometry as a single, continuous polyline approximating the original elevation contours. The start and end of the polyline are fitted with an entry and exit point for the end-effector to begin and end the toolpath (Supplementary Fig. 6d). The final set of x-y-z coordinates are then formatted into Move Instructions in the relevant executable code along with the initially defined fabrication parameters (Supplementary Fig. 6e).

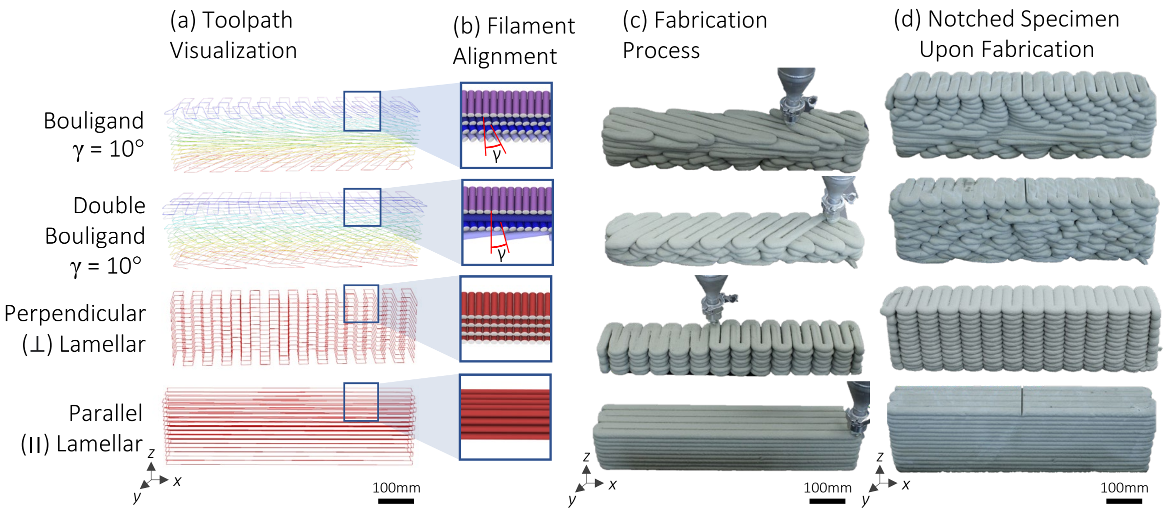

Supplementary Fig. 7. Test specimen toolpath design: (a) Toolpath visualization of the four architectures, (b) schematic diagram of filament arrangement of top four layers, highlighting the bouligand and double-bouligand pitch angle, (c) Fabrication process of each architecture, (d) notching of the test specimens upon fabrication and curing.

<u>Supplementary Note 7:</u> Specimen Notching and Preparation

Fabricated specimens were notched using a 16 mm tall by 0.2 mm thin aluminum strip that was inserted into the top of each beam precisely at the center, and depending on the architecture it was inserted between the two center filaments of the layer, and removed after an hour of being inserted. Each beam was fabricated with the architecture reversed such that the notch could be inserted from the top, in the bottommost layer of the testing orientation (Supplementary Fig. 7d). Prior to testing, the notch was meticulously sharpened using a bimetallic saw blade which was inserted such that the notch extended precisely to the end of the first layer. This placement leads to a continuous sharp notch through the width of the sample. The final, notched specimens are illustrated in Supplementary Fig. 7d. In the double-bouligand, bouligand, and perpendicular cases, the notch was introduced at the interfaces between two filaments, whereas in the parallel case, the notch was introduced perpendicular to the interfaces and filaments as shown in Supplementary Fig. 7d.

The beams were loaded on a universal testing unit under a displacement-controlled rate of 0.05 mm/min [43]. A loading rate of 0.05 mm/min leads to a time to reach peak load of 592 ± 156 seconds, which falls within the conventional range of loading rates [44,45]. More specifically, the load rate of 0.05 mm/min is within the typical range of the loading rate reported in literature [46–51].

<u>Supplementary Note 8</u>: Calculation of Mechanical Properties

In this study, the flexural strength and fracture toughness were characterized using three-point bend (3PB) tests and single-edge notched bend (SENB) tests [52,53], respectively.

Calculation of Modulus. The modulus of rupture was calculated per ASTM C293M-16 [54], based on the maximum peak load, P_{max} of the unnotched beam specimens:

$$MOR = \frac{3(P_{max})L}{2bd^2} \tag{1}$$

Where *L*, *b*, and *d* denote the testing span, beam width, and beam depth, respectively.

Calculation of Fracture Toughness. Fracture toughness calculations were made in accordance with ASTM E1820-20b [55]. The critical stress intensity factor, K_{Ic}, was calculated as the fracture toughness at the onset of crack propagation [52,53,56,57]. The fracture was assumed to initiate under Mode-I loading conditions and occurs at the maximum peak load:

$$K_{Ic} = \frac{P_{max}L}{bd^{1.5}} * f\left(\frac{a_o}{d}\right) \tag{2}$$

The function, $f\left(\frac{a}{d}\right)$, relates the crack tip extension, a, to the stress applied to a crack tip under single edge notched bending (SENB) conditions, for a 1:4 beam depth, d, to span, L, ratio. The crack tip length, a_o, was taken as the initial notch length.

$$\mathrm{f}\left(\frac{a_o}{d}\right) = \frac{3\left(\frac{a_o}{d}\right)^{\frac{1}{2}}(1.99-\left(\frac{a_o}{d}\right)\left(1-\frac{a_o}{d}\right)\left[2.15-3.93*\left(\frac{a_o}{d}\right)+2.7\left(\frac{a_o}{d}\right)^2\right])}{2\left(1+\frac{2a_o}{d}\right)\left(1-\frac{a_o}{d}\right)^{\frac{3}{2}}} \tag{3}$$

The *J*-integral is a mathematical expression which characterizes the stress field around the crack tip [55]. The calculation of the *J*-integral was computed as the summation of two parts: the elastic component of the *J*-integral, J_{el}, and the plastic component of the *J*-integral, J_{pl}.

$$J_{el} = \frac{K_{Ic}^2(1-v^2)}{E} \tag{4}$$

Compressive tests were performed on 7-day cylinders as per ASTM C39 [58] and the measured 7-day [59]elastic modulus, *E*, is 25.59 GPa. The Poisson's Ratio, v, was taken as 0.27 [60]. The K_{Ic} value was computed with a_o equal to the initial notch length. The plastic component of the *J*-integral was computed with the following ASTM formulation:

$$J_{pl} = \frac{\eta_{pl}A_{pl}}{b(d-a_0)} \tag{5}$$

Where for load-line displacement, η_{pl} is equal to 1.9, a_0 denotes the initial crack tip extension equivalent to the notch length, and A_{pl} was computed as the area under the force-displacement curve past the peak load of the specimen:

$$A_{pl} = \sum_{i=P_{max}}^{n} P_i \times \delta_i \tag{6}$$

The fracture toughness, K_{jc}, can then be computed as follows [61,62]:

$$K_{Jc} = \sqrt{(J_{el} + J_{pl})E} \tag{7}$$

The K_{Jc} curve (Fig. 3d) was computed for incremental points of crack extension, a_n, based on the instantaneous specimen compliance, C_n. Compliance was evaluated as the instantaneous ratio of crosshead displacement to applied load [63]:

$$C_n = \frac{u_n}{f_n} \tag{8}$$

Where u_n represents the instantaneous crosshead displacement for measurement *n*, and f_n represents the instantaneous load. Both crosshead and load measurements are taken at a frequency of 10 Hz. The instantaneous crack tip extension, a_n, was subsequently calculated as the following relationship [63]:

$$a_n = a_{n-1} + \frac{d-a_n}{2}\frac{C_n - C_{n-1}}{C_n} \tag{9}$$

As the crack tip extends, the A_{pl} increases and the evolution of K_{Jc} can be plotted against crack extension a_n as resistance curve (R-curve). The R-curve is a commonly used for understanding the fracture toughness of a material and is advantageous for concurrently investigating the material's toughness crack initiation (K_{Ic}) and crack propagation (K_{Jc}). The R-curves are plotted past the maximum crack extension of $a_{max} = 0.25(d - a_o)$ specified by ASTM E1820-20b (Fig. 3d) and are considered for the calculation of the *J*-integral [55]. For specimens exhibiting a post-peak softening, the toughness is measured for a crack extension corresponding to 1% of the respective specimen's peak load.

Calculation of Work of Fracture. The work of fracture represents the total energy required to generate a unit fracture area and was calculated as the total area under the load-displacement curve divided by two times the fractured surface area [43]:

$$\gamma_{WOF} = \sum_{i=1}^{n} \frac{P_i \times \delta_i}{b(d-a_o)} \tag{10}$$

Where P_i represents the instantaneous measured load and δ_i represents the corresponding instantaneous measured displacement.

<u>Supplementary Note 9</u>: Fresh and Hardened Properties of Cementitious Materials

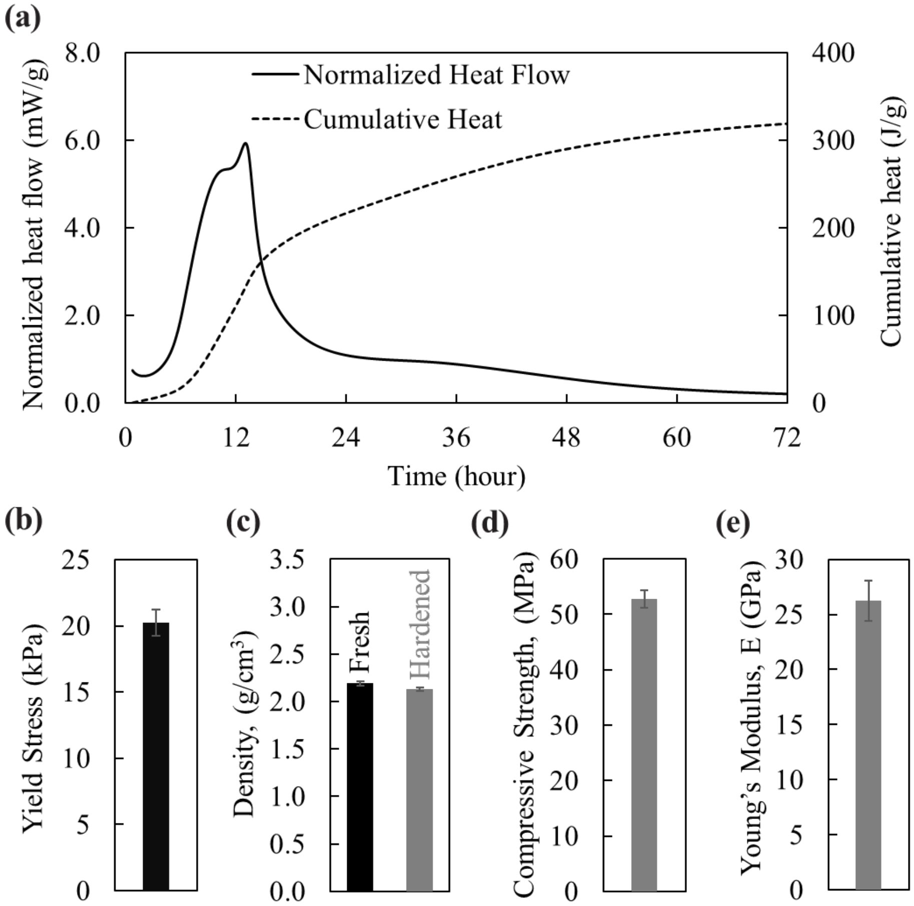

Supplementary Fig. 8. (a) Heat flow and cumulative heat, normalized to the weight of cement, obtained from isothermal calorimetry, (b) Yield stress of fresh concrete obtained from cone penetration test (CPT), (c) Density of fresh concrete and 7-day age hardened concrete, (d,e) 7-day compressive strength and Young's modulus of hardened concrete. Data is shown as mean ± SD.

The isothermal calorimeter analysis was performed using TAM Air ® to study the hydration kinetics of the concrete obtained from 2K system. Six samples of 25.728 g were kept at the bath temperature of 23°C and the heat flow and cumulative heat were recorded. The heat flow and cumulative heat, normalized with mass of cement, for a representative sample is presented in Supplementary Fig. 8a. The peak heat rate in the samples was found to be $6.573 \pm 0.611\ mW/g$ 13 hours after mixing which is similar to peak heat flow rate of $6.158 \pm 0.144\ mW/g$ after 14 – 15 hours in concrete with an aluminium sulfate-based accelerator with similar w/c (0.42) found in the literature [64].

The yield stress of fresh state concrete was performed using the cone penetration test on an additively manufactured prismatic specimen of 250 mm in length, 162 mm in width, and 65 mm in height (equivalent to 5 layers). The cone of diameter 20 mm and height 30 mm, connected to the cylinder of 20 mm diameter and 20 mm height, was used to penetrate the sample. The cone penetration test was

conducted with a displacement rate of 0.5 mm/s, and was carried out until the penetration load reached a plateau [65]. The printing time of each prismatic sample was 3 min, to which 4 min was adding for testing, hence leading to a total 7 min duration between the extrusion from the nozzle and end of the test. The static yield stress, τ_0, was calculated using the following equation [65,66]:

$$\tau_0 = \frac{P_{max}}{\pi R(\sqrt{R^2+h_2^2} + 2h)} \tag{11}$$

where, P_{max} is the maximum penetration load, R is the cone radius, h_2 is the cone height, and h is the height of penetrated part of the cylinder. The yield stress, τ_0, is found to be $20.2 \pm 1.0\ kPa$ (as shown in Supplementary Fig. 8b), which is similar to the range of yield stress (22.7 kPa, taken at 7 min for comparison) reported from cone penetration test of mortar with an aluminate-based accelerator [67].

The density of fresh and 7-day hardened concrete $2.193 \pm 0.023\ g/cm^3$ and $2.128 \pm 0.017\ g/cm^3$, respectively, as exhibited in Supplementary Fig. 8c. Furthermore, the bulk density of the 3D-printed samples decreased by 8.63%, 11.65%, 8.00%, and 7.12% for parallel, perpendicular, bouligand, and double bouligand samples, respectively, compared to bulk density of the cast sample.

The compressive strength of the 7-day hardened concrete was obtained using the compression testing of cylindrical samples (76.2 mm diameter and 152.4 mm height) according to ASTM C39 [68]. The displacement rate of the test was kept as 0.6 mm/min to maintain the stress rate of $0.2 \pm 0.05\ MPa/s$ as per the guidelines of the ASTM C39 [68]. Furthermore, by using the axial extensometer on cylindrical specimens of compression testing, the axial strain was recorded. The compressive stress and axial strain were then used to evaluate the Young's modulus of the concrete. The compressive strength and Young's modulus were found to be $52.73 \pm 1.62\ MPa$ and $25.59 \pm 1.72\ GPa$ as displayed in Supplementary Fig. 8d and 8e, respectively.

Supplementary Videos

Supplementary Video 1: Robotic additive manufacturing of concrete with double-bouligand architecture. Video link: https://youtu.be/0dNXpa5TkyE

Supplementary Video 2: Robotic additive manufacturing of concrete with bouligand architecture. Video link: https://youtu.be/hpeONS0q5qU

Supplementary Video 3: Robotic additive manufacturing of concrete with lamellar (perpendicular) architecture. Video link: https://youtu.be/qEQX4gH58Ow

Supplementary Video 4: Robotic additive manufacturing of concrete with lamellar (parallel) architecture. Video link: https://youtube.com/shorts/i80Kpts1wZI

Supplementary Video 5: Robotic additive manufacturing of architected concrete hollow column (first). Video link: https://youtube.com/shorts/xM5muDlL-6A?feature=share

Supplementary Video 6: Robotic additive manufacturing of architected concrete hollow column (second). Video link: https://youtube.com/shorts/IsmFldZQ4Hg

Supplementary Video 7: Robotic additive manufacturing of architected concrete hollow column (second, time lapse). Video link: https://youtu.be/efNKmO8MVpg

Supplementary Video 8: Robotic additive manufacturing of non-planar shell (vault) concrete. Video link: https://youtube.com/shorts/QrwgGRkTRE0?feature=share

Supplementary Video 9: Robotic additive manufacturing of helically architected component. Video link: https://youtu.be/JRZhpovHDyA

Supplementary Video 10: Robotic additive manufacturing of concrete with Hilbert architecture. Video link: https://youtube.com/shorts/uEZ-veFj390?feature=share

References

[1] R. Moini, J. Olek, P.D. Zavattieri, J.P. Youngblood, Early-age buildability-rheological properties relationship in additively manufactured cement paste hollow cylinders, Cem Concr Compos 131 (2022). https://doi.org/10.1016/j.cemconcomp.2022.104538.

[2] S.Z. Jones, D.P. Bentz, N.S. Martys, W.L. George, A. Thomas, Rheological control of 3D printable cement paste and mortars, in: RILEM Bookseries, Springer Netherlands, 2019: pp. 70–80. https://doi.org/10.1007/978-3-319-99519-9_7.

[3] S.A.O. Nair, G. Sant, N. Neithalath, Mathematical morphology-based point cloud analysis techniques for geometry assessment of 3D printed concrete elements, Addit Manuf 49 (2022) 102499. https://doi.org/https://doi.org/10.1016/j.addma.2021.102499.

[4] I. Ivanova, E. Ivaniuk, S. Bisetti, V.N. Nerella, V. Mechtcherine, Comparison between methods for indirect assessment of buildability in fresh 3D printed mortar and concrete, Cem Concr Res 156 (2022). https://doi.org/10.1016/j.cemconres.2022.106764.

[5] A.E. Douba, P. Badjatya, S. Kawashima, Enhancing carbonation and strength of MgO cement through 3D printing, Constr Build Mater 328 (2022). https://doi.org/10.1016/j.conbuildmat.2022.126867.

[6] Y. Chen, S. He, Y. Zhang, Z. Wan, O. Çopuroğlu, E. Schlangen, 3D printing of calcined clay-limestone-based cementitious materials, Cem Concr Res 149 (2021). https://doi.org/10.1016/j.cemconres.2021.106553.

[7] S. Muthukrishnan, S. Ramakrishnan, J. Sanjayan, Effect of alkali reactions on the rheology of one-part 3D printable geopolymer concrete, Cem Concr Compos 116 (2021) 103899. https://doi.org/10.1016/j.cemconcomp.2020.103899.

[8] D.P. Bentz, S.Z. Jones, I.R. Bentz, M.A. Peltz, Chapter 15 - Towards the Formulation of Robust and Sustainable Cementitious Binders for 3D Additive Construction by Extrusion, in: J.G. Sanjayan, A. Nazari, B. Nematollahi (Eds.), 3D Concrete Printing Technology, Butterworth-Heinemann, 2019: pp. 307–331. https://doi.org/https://doi.org/10.1016/B978-0-12-815481-6.00015-4.

[9] D. Marchon, S. Kawashima, H. Bessaies-Bey, S. Mantellato, S. Ng, Hydration and rheology control of concrete for digital fabrication: Potential admixtures and cement chemistry, Cem Concr Res 112 (2018) 96–110. https://doi.org/10.1016/j.cemconres.2018.05.014.

[10] L. Reiter, T. Wangler, A. Anton, R.J. Flatt, Setting on demand for digital concrete – Principles, measurements, chemistry, validation, Cem Concr Res 132 (2020) 106047. https://doi.org/10.1016/j.cemconres.2020.106047.

[11] F.B. Rodriguez, J. Olek, R. Moini, P.D. Zavattieri, J.P. Youngblood, Linking Solids Content and Flow Properties of Mortars to their Three-Dimensional Printing Characteristics, ACI Mater J 118 (2021) 371–382. https://doi.org/10.14359/51733136.

[12] N. Roussel, G. Ovarlez, S. Garrault, C. Brumaud, The origins of thixotropy of fresh cement pastes, Cem Concr Res 42 (2012) 148–157. https://doi.org/10.1016/j.cemconres.2011.09.004.

[13] N. Roussel, A thixotropy model for fresh fluid concretes: Theory, validation and applications, Cem Concr Res 36 (2006) 1797–1806. https://doi.org/10.1016/j.cemconres.2006.05.025.

[14] A. Kazemian, B. Khoshnevis, Real-time extrusion quality monitoring techniques for construction 3D printing, Constr Build Mater 303 (2021). https://doi.org/10.1016/j.conbuildmat.2021.124520.

[15] T.T. Le, S.A. Austin, S. Lim, R.A. Buswell, A.G.F. Gibb, T. Thorpe, Mix design and fresh properties for high-performance printing concrete, Materials and Structures/Materiaux et Constructions 45 (2012) 1221–1232. https://doi.org/10.1617/s11527-012-9828-z.

[16] V.N. Nerella, M. Krause, V. Mechtcherine, Direct printing test for buildability of 3D-printable concrete considering economic viability, Autom Constr 109 (2020) 102986. https://doi.org/https://doi.org/10.1016/j.autcon.2019.102986.

[17] G. Ma, R. Buswell, W.R. Leal da Silva, L. Wang, J. Xu, S.Z. Jones, Technology readiness: A global snapshot of 3D concrete printing and the frontiers for development, Cem Concr Res 156 (2022) 106774. https://doi.org/10.1016/j.cemconres.2022.106774.

[18] R.J.M. Wolfs, A.S.J. Suiker, Structural failure during extrusion-based 3D printing processes, International Journal of Advanced Manufacturing Technology 104 (2019) 565–584. https://doi.org/10.1007/s00170-019-03844-6.

[19] R.J.M. Wolfs, F.P. Bos, T.A.M. Salet, Early age mechanical behaviour of 3D printed concrete: Numerical modelling and experimental testing, Cem Concr Res 106 (2018) 103–116. https://doi.org/10.1016/j.cemconres.2018.02.001.

[20] W.R. Leal da Silva, H. Fryda, J.N. Bousseau, P.A. Andreani, T.J. Andersen, evaluation of early-age concrete structural build-up for 3D concrete printing by oscillatory rheometry, in: Advances in Intelligent Systems and Computing, Springer Verlag, 2020: pp. 35–47. https://doi.org/10.1007/978-3-030-20216-3_4.

[21] T. Wangler, R. Pileggi, S. Gürel, R.J. Flatt, A chemical process engineering look at digital concrete processes: critical step design, inline mixing, and scaleup, Cem Concr Res 155 (2022) 106782. https://doi.org/10.1016/j.cemconres.2022.106782.

[22] F.P. Bos, C. Menna, M. Pradena, E. Kreiger, W.R.L. da Silva, A.U. Rehman, D. Weger, R.J.M. Wolfs, Y. Zhang, L. Ferrara, V. Mechtcherine, The realities of additively manufactured concrete structures in practice, Cem Concr Res 156 (2022). https://doi.org/10.1016/j.cemconres.2022.106746.

[23] T. Wangler, E. Lloret, L. Reiter, N. Hack, F. Gramazio, M. Kohler, M. Bernhard, B. Dillenburger, J. Buchli, N. Roussel, R. Flatt, Digital Concrete: Opportunities and Challenges, RILEM Technical Letters 1 (2016) 67. https://doi.org/10.21809/rilemtechlett.2016.16.

[24] N. Roussel, Rheological requirements for printable concretes, Cem Concr Res 112 (2018) 76–85. https://doi.org/10.1016/j.cemconres.2018.04.005.

[25] R. Moini, J. Olek, P.D. Zavattieri, J.P. Youngblood, Early-age buildability-rheological properties relationship in additively manufactured cement paste hollow cylinders, Cem Concr Compos 131 (2022) 104538.

[26] R. Moini, A. Baghaie, F.B. Rodriguez, P.D. Zavattieri, J.P. Youngblood, J. Olek, Quantitative microstructural investigation of 3D-printed and cast cement pastes using micro-computed tomography and image analysis, Cem Concr Res 147 (2021) 106493.

[27] A. Douba, S. Ma, S. Kawashima, Rheology of fresh cement pastes modified with nanoclay-coated cements, Cem Concr Compos 125 (2022) 104301.

[28] A. Douba, C. Chan, S. Berrios, S. Kawashima, Synthesis of hybridized rheological modifiers for 3D concrete printing, in: Second RILEM International Conference on Concrete and Digital Fabrication: Digital Concrete 2020 2, Springer, 2020: pp. 32–41.

[29] A. Das, L. Reiter, S. Mantellato, R.J. Flatt, Early-age rheology and hydration control of ternary binders for 3D printing applications, Cem Concr Res 162 (2022) 107004.

[30] D. Marchon, S. Kawashima, H. Bessaies-Bey, S. Mantellato, S. Ng, Hydration and rheology control of concrete for digital fabrication: Potential admixtures and cement chemistry, Cem Concr Res 112 (2018) 96–110.

[31] T. Wangler, R. Pileggi, S. Gürel, R.J. Flatt, A chemical process engineering look at digital concrete processes: critical step design, inline mixing, and scaleup, Cem Concr Res 155 (2022) 106782.

[32] N. Roussel, Rheological requirements for printable concretes, Cem Concr Res 112 (2018) 76–85.

[33] A. Zaheri, J.S. Fenner, B.P. Russell, D. Restrepo, M. Daly, D. Wang, C. Hayashi, M.A. Meyers, P.D. Zavattieri, H.D. Espinosa, Revealing the mechanics of helicoidal composites through additive manufacturing and beetle developmental stage analysis, Adv Funct Mater 28 (2018) 1803073.

[34] T.M. Inc., MATLAB version: 9.13.0 (R2022b), (2022). https://www.mathworks.com.

[35] M. Moini, J. Olek, B. Magee, P. Zavattieri, J. Youngblood, Additive manufacturing and characterization of architectured cement-based materials via X-ray micro-computed tomography, in: RILEM Bookseries, Springer Netherlands, 2019: pp. 176–189. https://doi.org/10.1007/978-3-319-99519-9_16.

[36] R. Moini, A. Baghaie, F.B. Rodriguez, P.D. Zavattieri, J.P. Youngblood, J. Olek, Quantitative microstructural investigation of 3D-printed and cast cement pastes using micro-computed tomography and image analysis, Cem Concr Res 147 (2021). https://doi.org/10.1016/j.cemconres.2021.106493.

[37] M. van den Heever, A. du Plessis, J. Kruger, G. van Zijl, Evaluating the effects of porosity on the mechanical properties of extrusion-based 3D printed concrete, Cem Concr Res 153 (2022) 106695.

[38] M.K. Mohan, A. V Rahul, J.F. Van Stappen, V. Cnudde, G. De Schutter, K. Van Tittelboom, Assessment of pore structure characteristics and tortuosity of 3D printed concrete using mercury intrusion porosimetry and X-ray tomography, Cem Concr Compos 140 (2023) 105104.

[39] H. Lee, J.-H.J. Kim, J.-H. Moon, W.-W. Kim, E.-A. Seo, Correlation between pore characteristics and tensile bond strength of additive manufactured mortar using X-ray computed tomography, Constr Build Mater 226 (2019) 712–720.

[40] J. Kruger, A. du Plessis, G. van Zijl, An investigation into the porosity of extrusion-based 3D printed concrete, Addit Manuf 37 (2021) 101740.

[41] S. Gupta, H.S. Esmaeeli, A. Prihar, R.M. Ghantous, J.W. Weiss, R. Moini, Heterogeneity in 3D-Printed Lamellar Cementitious Materials: A Microstructure, Fracture, and Transport Analysis, (n.d.). https://doi.org/Unpublished Results.

[42] R. McNeel, others, Rhinoceros 3D, Version 6.0, Robert McNeel & Associates, Seattle, WA (2010).

[43] M. Sakai, H. Ichikawa, Work-of-fracture of brittle materials with microcracking and crack bridging, Int J Fract 55 (1992) 65–79. https://doi.org/10.1007/BF00018033.

[44] C.G. Hoover, Z.P. Bažant, J. Vorel, R. Wendner, M.H. Hubler, Comprehensive concrete fracture tests: Description and results, Eng Fract Mech 114 (2013) 92–103.

[45] Z.P. Bazant, R. Gettu, Rate effects and load relaxation in static fracture of concrete, ACI Mater J 89 (1992) 456–468.

[46] Y. Ruan, B. Han, X. Yu, Z. Li, J. Wang, S. Dong, J. Ou, Mechanical behaviors of nano-zirconia reinforced reactive powder concrete under compression and flexure, Constr Build Mater 162 (2018) 663–673.

[47] M. Sakai, H. Ichikawa, Work-of-fracture of brittle materials with microcracking and crack bridging, Int J Fract 55 (1992) 65–79.

[48] J. Zhang, W. Dong, B. Zhang, Experimental study on local crack propagation of concrete under three-point bending, Constr Build Mater 401 (2023) 132699.

[49] L. Qing, G. Cao, J. Guan, S. Li, Experimental method for determining the fracture toughness of concrete based on the modified two-parameter model and DIC technique, Fatigue Fract Eng Mater Struct 45 (2022) 400–410.

[50] Y. Yin, Y. Qiao, S. Hu, Four-point bending tests for the fracture properties of concrete, Eng Fract Mech 211 (2019) 371–381.

[51] J. Xie, Y. Liu, M.-L. Yan, J.-B. Yan, Mode Ⅰ fracture behaviors of concrete at low temperatures, Constr Build Mater 323 (2022) 126612.

[52] Z.P. Bazant, J. Planas, Fracture and size effect in concrete and other quasibrittle materials, Routledge, 2019.

[53] Y. Jenq, S.P. Shah, Two parameter fracture model for concrete, J Eng Mech 111 (1985) 1227–1241.

[54] ASTM International, C293/C293M-16 Standard Test Method for Flexural Strength of Concrete (Using Simple Beam With Center-Point Loading), (2016). https://doi.org/10.1520/C0293_C0293M-16.

[55] ASTM International, E1820-21 Standard Test Method for Measurement of Fracture Toughness, (2021). https://doi.org/10.1520/E1820-21.

[56] J.E. Srawley, Wide range stress intensity factor expressions for ASTM E 399 standard fracture toughness specimens, in: Conf. of Am. Soc. for Testing and Mater., Committee E-24, 1976.

[57] H.-L. Gao, S.-M. Chen, L.-B. Mao, Z.-Q. Song, H.-B. Yao, H. Cölfen, X.-S. Luo, F. Zhang, Z. Pan, Y.-F. Meng, Mass production of bulk artificial nacre with excellent mechanical properties, Nat Commun 8 (2017) 287.

[58] ASTM International, Standard Test Method for Compressive Strength of Cylindrical Concrete Specimens, (n.d.). https://doi.org/10.1520/C0039_C0039M-21.

[59] American Concrete Institute, Guide for modeling and calculating shrinkage and creep in hardened concrete, American Concrete Institute, 2008.

[60] S. Harsh, Z. Shen, D. Darwin, Strain-Rate Sensitive Behavior of Cement Paste and Mortar in Compression, ACI Mater J (1990).

[61] F. Bouville, E. Maire, S. Meille, B. van de Moortèle, A.J. Stevenson, S. Deville, Strong, tough and stiff bioinspired ceramics from brittle constituents, Nat Mater 13 (2014) 508–514. https://doi.org/10.1038/nmat3915.

[62] A. Amini, A. Khavari, F. Barthelat, A.J. Ehrlicher, Centrifugation and index matching yield a strong and transparent bioinspired nacreous composite, Science (1979) 373 (2021) 1229–1234. https://doi.org/10.1126/science.abf0277.

[63] F. Bouville, E. Maire, S. Meille, B. Van de Moortèle, A.J. Stevenson, S. Deville, Strong, tough and stiff bioinspired ceramics from brittle constituents, Nat Mater 13 (2014) 508–514.

[64] A. Szabo, L. Reiter, E. Lloret-Fritschi, F. Gramazio, M. Kohler, R.J. Flatt, Mastering yield stress evolution and formwork friction for smart dynamic casting, Materials 13 (2020) 2084.

[65] I. Ivanova, E. Ivaniuk, S. Bisetti, V.N. Nerella, V. Mechtcherine, Comparison between methods for indirect assessment of buildability in fresh 3D printed mortar and concrete, Cem Concr Res 156 (2022) 106764.

[66] D. Lootens, P. Jousset, L. Martinie, N. Roussel, R.J. Flatt, Yield stress during setting of cement pastes from penetration tests, Cem Concr Res 39 (2009) 401–408.

[67] F. Boscaro, E. Quadranti, T. Wangler, S. Mantellato, L. Reiter, R.J. Flatt, Eco-friendly, set-on-demand digital concrete, 3D Print Addit Manuf 9 (2022) 3–11.

[68] ASTM C39/C39M-23, Standard Test Method for Compressive Strength of Cylindrical Concrete Specimens, ASTM International (2023).
