# Supplementary material for: Tough double-bouligand architected concrete enabled by robotic additive manufacturing
Source: Nat Commun. 2024 Aug 29;15:7498. doi: 10.1038/s41467-024-51640-y (PMC11362293; doi:10.1038/s41467-024-51640-y)
Supplement: Supplementary file 3 — Description of Additional Supplementary Files [file 41467_2024_51640_MOESM3_ESM.pdf]

Description of Additional Supplementary Files

File Name: Supplementary Video 1
Description: Robotic additive manufacturing of concrete with double-bouligand architectures

File Name: Supplementary Video 2
Description: Robotic additive manufacturing of concrete with bouligand architecture

File Name: Supplementary Video 3
Description: Robotic additive manufacturing of concrete with lamellar (perpendicular) architecture

File Name: Supplementary Video 4
Description: Robotic additive manufacturing of concrete with lamellar (parallel) architecture

File Name: Supplementary Video 5
Description: Robotic additive manufacturing of architected concrete hollow column (first)

File Name: Supplementary Video 6
Description: Robotic additive manufacturing of architected concrete hollow column (second)

File Name: Supplementary Video 7
Description: Robotic additive manufacturing of architected concrete hollow column (second, time lapse)

File Name: Supplementary Video 8
Description: Robotic additive manufacturing of non-planar shell (vault) concrete

File Name: Supplementary Video 9
Description: Robotic additive manufacturing of helically architected component

File Name: Supplementary Video 10
Description: Robotic additive manufacturing of concrete with Hilbert architecture
